# Standardisation Strategies for Nursing Handovers in Paediatric Hospitalisation: A Scoping Review

**DOI:** 10.3390/nursrep16030084

**Published:** 2026-02-27

**Authors:** Pablo Buck Sainz-Rozas, Laia García Fernández, Marina Duque Domínguez

**Affiliations:** 1 Hospital Infantil i de la Dona, Vall d’Hebron Barcelona Hospital Campus, Pg. de la Vall d’Hebron, 119, Horta-Guinardó, 08035 Barcelona, Catalunya, Spain; 2 Instituto Español de Investigación Enfermera (IE), Calle Sierra de Pajarejo nº 13, 28023 Madrid, Madrid, Spain; 3 Departamento de Enfermería, Universidad Autónoma de Barcelona, Av. de Can Domènech, 737, 08193 Cerdanyola del Vallès, Barcelona, Spain

**Keywords:** patient safety, patient handover, communication, nurses, paediatrics, reference standards

## Abstract

**Background/Objectives**: To identify existing evidence on strategies for standardising nursing handovers in paediatric hospital settings, given their impact on communication, safety, and quality of care. International bodies such as the WHO and The Joint Commission recommend standardisation as a key measure to reduce patient safety incidents. **Methods**: A scoping review was conducted in December 2022 using Medline, Cochrane Library, Scopus, and CINAHL databases. The search strategy included documents published between 2012 and 2022, in Spanish, English, Catalan, French, and/or Portuguese. We screened according to inclusion criteria (professional nurses and hospitalisation) and exclusion criteria (intensive care and medical professionals) and tabulated the results according to concurrent themes. The PRISMA-ScR guidelines were followed. **Results**: A total of 308 records were identified. After screening, 25 full-text articles were assessed for eligibility. Following quality appraisal, six were excluded for not meeting predefined criteria, resulting in 19 studies included in the final synthesis. The evidence mapped shows that most structured communication tools have been developed or validated in adult or medical contexts, with limited evaluation in paediatric nurse-to-nurse inpatient settings. Standardised structured communication tools used in hospital settings include SBAR, I-PASS, and Flex 11, while assessment instruments such as the Handoff CEX Scale and Handover Evaluation Scale have been applied to evaluate handover quality. **Conclusions**: Structured communication tools may contribute to improving information transfer and perceived quality of handover; however, paediatric nurse-specific evidence remains limited and frequently derives from non-nursing or adult contexts. Further adaptation and validation in paediatric inpatient nursing settings are required.

## 1. Introduction

Patient safety is the reduction in adverse events associated with healthcare to the minimum acceptable risk [1]. Failures in communication during clinical processes, including patient handover, account for a substantial proportion of adverse and sentinel events internationally [2,3,4].

International patient safety strategies, including the WHO Global Patient Safety Action Plan 2021–2030, emphasise the critical importance of safe communication during transitions of care and information transfer (Strategic Objective 3) [5,6].

The forms of this transfer of information include patient handoff or patient handover. Cohen and Hilligoss define patient handover as the process of exchanging clinical information about patients between two or more professionals during a shift change, at the time of discharge or transfer of the patient. Handover involves transferring responsibility for a patient, including clinical aspects and legal liability, to another person [7], as noted by Elliot et al., who defined patient handoff as the process by which patient care is transferred from one professional to another [8].

The transfer of information at patient handovers is recognised as a high-risk event, being a frequent breach of safety, as it is estimated that more than 4000 information handovers occur in a typical university hospital every day [2,9]. In paediatric hospital settings, this process presents additional complexity due to age-related clinical variability, active family involvement, and the central role of nursing staff in continuous inpatient care. Recent qualitative evidence highlights that family participation in nursing bedside handover influences perceptions of communication quality and safety [10].

The quality of patient handover is primarily determined by communication effectiveness and may be compromised by message-related factors (e.g., imprecision, omissions, inappropriate jargon) [7,11,12,13], professional factors (e.g., communication skills, attitudes, multitasking, and ethical engagement) [2,14,15,16], and environmental conditions such as workload, time pressure, and inadequate physical settings [17,18].

In response to this relevant public health problem, in 2005, the WHO and the Joint Commission published Nine patient safety solutions, with ‘communication during patient handover’ being the third solution, in which it was recommended to implement a standardised system for communication between staff at the time of handover, shift changes, and in the event of transfer to other units [13].

Handover standardisation has been performed by different authors using templates to present information [15], with checklists [16], technological tools, standardised reports, or instruments with a minimum set of data to be provided [11], together with the training of students [19] and nursing professionals for its implementation [2,13].

In addition to transfer standardisation, it is necessary to structure the message, which has been considered a priority by several authors, as it favours the complete, effective, and collaborative transfer of information, limiting the likelihood of errors [11,12,15,20].

Moreover, it is emphasised that the structure and content of the patient handover should be specific to the unit in question or, in the case of being general, adaptable to different settings [12] while ensuring that the handover can be adapted to the clinical condition of the patient, the service in question, or the professional’s level of experience [11].

Additional approaches such as communication skills training, family involvement, and bedside handover have also been proposed to address barriers in paediatric contexts [7,21].

Although recent quasi-experimental studies have begun to evaluate structured handover interventions in paediatric inpatient nursing [22], the evidence remains dispersed and has not been comprehensively synthesised. Most available tools were developed in adult or physician-led contexts, and their applicability to paediatric nursing practice has not been comprehensively mapped. This lack of synthesis justifies the need for a scoping review to identify, categorise, and analyse the existing evidence.

Given this context, the objective of this scoping review was to map and synthesise existing evidence on strategies and tools aimed at standardising nurse-to-nurse patient handover in paediatric hospital settings [2,9].

In this review, ‘standardisation’ refers to the implementation of formalised protocols, checklists, mnemonics or electronic tools designed to ensure consistency in information transfer, whereas ‘structuring’ refers to the organisation and sequencing of message content during communication. Our focus is specifically on nurse-to-nurse shift-to-shift handovers in paediatric inpatient units.

The research question guiding this scoping review was: What evidence exists regarding strategies and tools for standardising nurse-to-nurse patient handover in paediatric inpatient hospital settings? Given the complexity and crosscutting nature of the topic, a broad scope of the review question was chosen.

## 2. Materials and Methods

A scoping review was conducted following the Preferred Reporting Items for Systematic Reviews and Meta-Analyses Scoping Review extension (PRISMA-ScR) guidelines and checklist to achieve the highest possible methodological rigour, within the limitations of the question posed [23]. The search protocol was registered in OSF Registries [24].

### 2.1. Sources of Information and Search Strategy

The search was conducted using the Cochrane Library tertiary information database and secondary sources: Medline, Scopus, and CINAHL. These databases were selected due to their comprehensive coverage of nursing, medical, and interdisciplinary healthcare literature. Scopus provides broad indexing of biomedical and health sciences journals.

The scientific literature review was conducted in December 2022. The search strategy for each database was outlined around two conceptual sets: ‘paediatric’ and ‘patient handover’. Each set was expanded as much as possible by incorporating a thesaurus (MeSH terms, Table 1). For the standardised terms, the concepts ‘shift-to-shift handover’ and ‘shift report*’ were added as natural terms, and in those databases that did not allow the use of thesauri, the search was performed with natural terms.

To refine the search, limits were applied by using different filters in the databases. Two filters, i.e., year of publication and language, were applied. To these results, we added those found using a snowball technique which, despite not being found in the search performed, are of relevance for the present review.

### 2.2. Inclusion and Exclusion Criteria

The inclusion and exclusion criteria used in this review are listed in Table 2.

### 2.3. Study Selection Process

The articles resulting from the search were allocated to reviewers based on their respective databases of origin. Each reviewer independently decided, based on the reading of titles and abstracts, the eligibility of each study based on the criteria described above and using three possible answers: yes, no, or unsure. A table was prepared for each database, including the title of the study, the eligibility assessment (yes/no/unsure), and the observations (justifying the decision). Next, a consensus was reached among the authors to decide whether those considered doubtful met the inclusion criteria of the review.

Finally, the full texts of the studies considered suitable for the review were obtained. Due to the final number of articles selected, it was decided to distribute them among the reviewers based on the differentiated conceptual sets. The review was conducted independently using a Mendeley reference manager as an automation tool to enable subsequent distribution of articles.

### 2.4. Tools for Assessing Quality and Risk of Bias

This process was carried out independently by each reviewer (PB, MD, LG) according to the previously described distribution of articles, and the articles were pooled to resolve any unclear aspects. Methodological quality was assessed using the tools of the Critical Appraisal Skills Programme Español (CASPe) [25,26,27] for qualitative studies, randomised clinical trials, and systematic reviews. Likewise, the 2018 version of the Mixed Methods Appraisal Tool (MMAT) was used to assess quasi-experimental and mixed studies [28]. For observational studies, the Strengthening The Reporting of OBservational studies in Epidemiology (STROBE) checklist [29] was used. The guidelines of each scale were followed to assess quality, scoring one if the item was in the article and 0 if it was absent. In all cases, a score equal to or greater than 7 out of 10 was considered valid based on prior methodological applications of these tools and consensus among the authors to ensure minimum reporting and internal validity standards. Although critical appraisal is not mandatory in scoping reviews, recent methodological guidance recognises that it may be incorporated when transparently justified. In this review, quality assessment was included descriptively to enhance transparency and contextual interpretation of findings, rather than to determine effectiveness or produce graded recommendations [29].

Studies scoring below the predefined threshold were excluded solely to ensure minimum reporting transparency and interpretability; however, methodological appraisal was not used to rank evidence hierarchically nor to inform effectiveness conclusions, in line with the exploratory purpose of this scoping review. For the articles included, the level of evidence was determined according to Sackett’s method, and the grade of recommendation according to a modified GRADE system to provide readers with a descriptive contextual framework regarding study design and evidentiary characteristics; these classifications were not used to generate practice recommendations or to imply effectiveness hierarchy. These classifications were not used to exclude studies but to contextualise the narrative synthesis and highlight where conclusions rely on higher- or lower-level evidence. Finally, the full text of each article was read by the reviewers.

### 2.5. Data Extraction and Synthesis

This was performed by each reviewer independently through full-text reading. An Excel table was created to record the data into previously agreed subtopics to facilitate subsequent analysis, and was completed by each author and subsequently reviewed by the rest. The information was grouped into the following topics: definition of ‘handover communication’, epidemiology on patient handover, patient safety-handover communication, professionals, barriers related to professionals, tools for assessing patient handover, tools for structuring patient handover, and best practices. The data extracted from the articles and classed in the table above were pooled and synthesised according to the structured thematic groups. During data extraction, studies were categorised according to population (paediatric vs. adult/mixed) and professional scope (nursing vs. medical/multiprofessional) to facilitate contextual interpretation of findings.

## 3. Results

### 3.1. Selection of Studies

**Figure 1:**
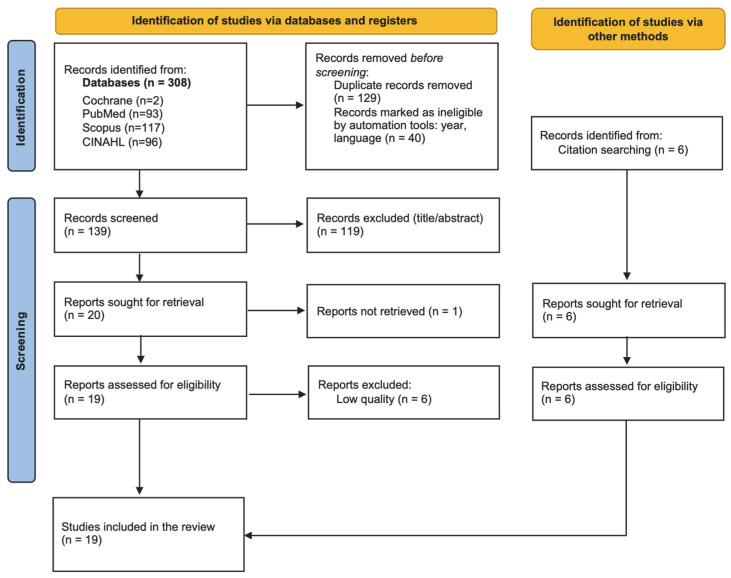
2020 PRISMA flow diagram of the search process.

The database search identified 308 records (93 from PubMed, 2 from the Cochrane Library, 117 from Scopus, and 96 from CINAHL), as shown in Figure 1. After applying year (n = 34) and language (n = 6) filters, 40 records were excluded, and 129 duplicates were removed.

Following title and abstract screening, 119 records were excluded for not meeting the eligibility criteria. One full-text article could not be retrieved. A total of 25 full-text articles were assessed for eligibility, including six identified through citation tracking.

### 3.2. Risk of Bias and Characteristics of the Studies

Of the 25 full-text articles assessed for eligibility, six studies did not achieve the predefined methodological threshold (≥7/10). In line with our intention to enhance the robustness of the interpretative synthesis—while maintaining the exploratory purpose of the scoping review—these studies were not included in the final narrative synthesis. The remaining 19 studies were included in the final synthesis.

Table 3 specifies, for each accepted study, the title, author, year of publication, type of study, grade of evidence based on Sackett’s method, grade of recommendation according to the GRADE system, and the score obtained in the 1–10 methodological quality appraisal.

### 3.3. Context of Evidence by Population and Professional Scope

Of the 19 included studies, 4 were conducted specifically in paediatric nurse-to-nurse inpatient handovers, 5 were undertaken in paediatric but primarily medical or multidisciplinary contexts, and 10 were conducted in adult or mixed settings, with findings extrapolated to paediatric nursing practice.

## 4. Discussion

### 4.1. Structured Communication Tools for Patient Handover

The evidence identified in this review derives from heterogeneous settings. Some studies were conducted specifically in paediatric nursing inpatient units, others in paediatric but primarily medical or multiprofessional contexts (e.g., resident handover), and several originated in adult or mixed populations. The applicability of each tool to paediatric nurse-to-nurse handovers is therefore discussed accordingly.

There are various evidence-supported initiatives and methods for standardising patient handover through structured communication tools. On the one hand, Lazzara et al. sought to determine if there were differences in attitudes, behaviours, and duration of the handover using the Flex 11 protocol versus the SBAR (Situation, Background, Assessment, and Recommendation). The results suggested that Flex 11, a tool consisting of 11 categories divided into cards, was effective and beneficial in a simulated setting, supporting the standardisation of handover by providing a predefined structure for information exchange [30]. Using Flex 11, practitioners had more positive attitudes, provided more information with the tool, and spent the same amount of time performing the handover independently [32].

On the other hand, SBAR is one of the most widely used methods across healthcare settings. However, most of the evidence supporting its effectiveness derives from adult or multiprofessional contexts, with limited paediatric nurse-specific outcome data. It consists of 4 items: Situation, Background, Assessment, and Recommendation. The ISBAR (Identify–Situation–Background–Assessment–Recommendation), SBAR-R (Situation–Background–Assessment–Recommendation–Readback), ISBARR (Identify–Situation–Background–Assessment–Recommendation–Readback), and ISOBAR (Identify–Situation–Observation–Background–Assessment–Recommendation) tools are structured adaptations derived from the original SBAR framework. The use of this strategy facilitates both inter- and intra-professional communication [20], ensuring that vital information is not lost, and that delivery can be done in an organised, timely, efficient, logical manner and at bedside [32].

Units that used the SBAR method and performed patient handover at a specific location obtained better results in terms of organisation. Müller et al. argued that preparation was required before use, as both the issuer and receiver had to share the same mental model. Furthermore, it is a communication technique that has been associated with improvements in communication processes and patient safety outcomes, and is widely regarded as a reference framework for transmitting information in critical situations and preventing adverse events [7,20,32].

The authors of this review studied the influence of the SBAR method on patient safety by calculating the incidence of adverse events. They concluded that SBAR implementation was associated with improvements in patient-related outcomes in predominantly adult and multidisciplinary contexts [20], with limited direct evidence in paediatric nurse-to-nurse inpatient settings. Professional satisfaction improved with the implementation of this tool and was related to a better perception of interdisciplinary communication and teamwork. The authors concluded that the SBAR tool can be adapted to multiple health services, especially those in which clear and effective communication between professionals is required [7,20,32].

In parallel, another tool that can be useful for information transfer is I-PASS (Illness severity–Patient summary–Action list–Situation awareness and contingency planning–Synthesis by receiver) [31]. This mnemonic framework is used to standardise oral and written information transfers by prescribing a structured sequence for message delivery. This includes severity of illness, a patient summary, an action list, situational awareness, and contingency plans. Its implementation was associated with a relative reduction in the rate of medical errors in paediatric resident populations. Direct evidence in paediatric nurse-to-nurse handovers remains limited [18,31].

Along the same lines, O’toole et al. concurred on the acquisition of fundamental patient care skills by participants and competently distinguished between high- and low-quality handover using the I-PASS handover assessment tools. This technique has been associated with improvements in safety, efficiency, and effectiveness of handover primarily in paediatric resident populations, with limited direct evaluation in paediatric nurse-to-nurse settings [2].

Other standardised structured communication approaches include Nursing Bedside Shift Report (NBSR) and SAFETIPS (Statistics–Assessment–Focused plan–Exam findings–To dos–If/thens–Pointers/Pitfalls–Severity of illness) mnemonic charts, which help in transferring patients’ clinical records between shifts, maintaining patient safety, reducing risk of harm, and improving the perception of handover and communication [21,33]. These approaches aim to reduce variability in handover by combining process standardisation with explicit structuring of communication content. The main structuring tools used in the search are shown in Table 4.

### 4.2. Assessment of Patient Handovers

Tools have been described in the literature to assess the quality of nursing patient handover, with different methodologies to evaluate its characteristics, such as the environment in which it takes place, the information provided, the time invested, or the personal perception [7,12,13].

The Handover Evaluation Scale (HES) tool is used to assess the quality of patient handover. Brown et al. examined the quality of handover in neonatal units and collected demographic information in relation to the quality of information, interaction and support, efficiency, and parental inclusion [12]. The authors concluded that the HES tool is a valid measure for monitoring and assessing the transfer process. The application of this scale resulted in the transmission of high-quality information, which the authors linked to the use of each professional’s particular methods for the handover, such as the SBAR tool or the use of electronic media [12].

Another tool for assessing the quality and characteristics of the handover is the Handoff CEX Scale, which has been validated for use in English and Italian. This scale assesses location, organisation, communication skills, content, clinical judgment, and humanistic and professional qualities. The scale also features a second phase, which is to be performed by the information recipient, which includes the same number of items but with different content. Each item was rated on a Likert scale ranging from 1 to 9 [7].

Yáñez-Corral et al. also studied how to assess the quality of patient handover [13]. In this case, they developed a measurement instrument with the aim of improving safety, avoiding repetitions, and work overload, while making it adaptable to different populations based on patient types. Sociodemographic data, structure of the patient handover (time, place, etc.), the process to be followed (interventions, procedures, examinations, and pending tests), and observations were included. The authors concluded that the instrument is valid and reliable for use in similar settings. When applying this scale to their study population, Brown et al. concluded that the quality of the handover was not adequate due to the amount of relevant information lost in the process [13].

In other studies, the authors assessed handover quality by direct observation, transfer of documents and recordings of oral handovers, or self-administered Likert scales [2,18,31,33]. In some cases, these scales were administered before and after a patient handover quality improvement intervention, which helped to assess participants’ perceived quality of the handover process [33].

Overall, the applicability of assessment tools to paediatric nurse-to-nurse inpatient settings varies. The HES has been applied in neonatal nursing and may be useful for evaluating communication quality and parental inclusion [12], whereas the Handoff CEX appears more suitable for structured observational assessment in broader or multidisciplinary contexts [7]. The Yáñez-Corral instrument offers a structured evaluation framework but may require contextual adaptation for paediatric inpatient nursing [13]. The limited number of paediatric nursing-specific validation studies underscores the need for further research in this area.

### 4.3. Limitations

Firstly, the main limitation of our study may be that the results can only be applicable to the paediatric setting, as most studies took an adult-derived approach without a methodologically acceptable validation. A limited proportion of the included studies evaluated tools exclusively within paediatric nurse-to-nurse inpatient handovers. Consequently, some recommendations rely on extrapolation from paediatric medical or adult populations, which may affect external validity.

Secondly, the participation of only four authors in the review may have limited the diversity of perspectives and completeness of the data analysis, despite articles being read and assessed independently. Thirdly, a limited number of databases were selected to enhance operability, which could have restricted access to a wider range of relevant studies. We sought to resolve this by using the major databases and broadening the scope of the research question. Fourthly, the broad scope of the review question may also have made it difficult to focus on specific aspects of patient handover in paediatric nursing, affecting the depth of the results. Although this review differentiates conceptually between standardisation and structuring, several included studies used these terms interchangeably, which may reflect broader conceptual ambiguity in the literature.

Finally, the methodological limitations of some included studies may have affected the overall robustness of the synthesis of results may have negatively affected the validity and reliability of the review, which should be considered when interpreting the study findings and conclusions.

### 4.4. Applicability

Our results were grouped by the main existing tools for standardising patient handover in paediatric hospitalisation. The implementation of these tools may contribute to improvements in the quality of patient handover and nurses’ satisfaction with the communication process, and may help reduce communication errors, particularly when adapted to paediatric nursing contexts, thereby potentially enhancing patient safety.

In turn, the available tools for assessing the quality of patient handover may support the identification of deficiencies in the transfer process and facilitate baseline assessment, although only a limited number have been specifically validated in paediatric nurse-to-nurse inpatient settings. In addition, these tools enable different professionals, environments, and moments in time to be compared, while also paving the way to continuous monitoring through audit programs.

As future lines of research, further studies are needed to adapt the tools associated with adult patient handover to the paediatric setting, but also to agree and validate their content and effectiveness, which may require experimental studies.

Due to the different places of publication of most of the existing tests, it is necessary to translate the retrieved instruments and tools into different languages to promote their use and the design of implementation studies. In addition, they are to be adapted and validated to the context of each unit.

Finally, new instruments based on the latest evidence, expert consensus, and content validation should be developed to address the specific needs of certain units and/or their characteristics.

## 5. Conclusions

Based on the findings of this scoping review of standardisation strategies for patient handover in paediatric nursing, the available evidence suggests that there is a need to focus on the continuous improvement of communication during this crucial process in healthcare. Despite the methodological limitations identified in the reviewed studies and the diversity of approaches taken in them, implementing structured communication tools—particularly those evaluated in paediatric nursing contexts—may enhance information transfer. However, the current evidence base specific to paediatric nurse-to-nurse handovers remains limited and warrants further high-quality research.

In addition to contributing to the reduction in potential miscommunication, this approach may contribute to efforts aimed at enhancing patient safety, boosting job satisfaction among nursing professionals, and ultimately, improving the quality of care provided to paediatric patients.

Against this backdrop, there is an opportunity to further strengthen structured handover practices in paediatric nursing, which encourages professionals to actively explore new avenues for the development and validation of specific structuring and standardisation tools.

The development of innovative tools supported by scientific evidence and the consensus of the nursing community has the potential to improve the quality of care provided to paediatric patients, but also may contribute to the continuous improvement of patient safety and the effectiveness of nursing care in the hospital setting.

## Figures and Tables

**Table 1:** PubMed search strategy.

Search Strategy
(“pediatrics”[MeSH] OR “pediatric nursing”[MeSH] OR “hospitals, pediatric”[MeSH]) AND (“patient handoff”[MeSH] OR “shift-to-shift handover”[Title/Abstract] OR “shift report*”[Title/Abstract])

* indicates the truncation of the final term in order to capture all possible word variants derived from the same root.

**Table 2:** Inclusion and exclusion criteria.

Inclusion Criteria	Exclusion Criteria
Publications in Spanish, English, Catalan, Portuguese, or French.	Studies conducted exclusively in low-resource settings.
Publications between 2012 and 2022.	Non-evidence-based recommendations from societies.
Studies analysing paediatric patient handover.	Grey literature (brochures, editorials, posters, and reports).
Studies analysing communication related to the clinical safety of paediatric patients.	Clinical practice guidelines, protocols, and non-evidence-based tools.
Studies analysing nurse-to-nurse communication in paediatric settings.	Studies focusing exclusively on highly specialised units (e.g., critical care, emergency, operating theatre, neonatal intensive care).
Studies that provide or include tools for information transfer among paediatric nurses.	Studies focusing on inter-hospital referral or continuity of care between different institutions.
Studies that provide or include tools for the assessment of information transfer among paediatric nurses.	Studies focusing on the referral of patients from different hospitals.
Randomised or non-randomised clinical trials on the study subject.	Studies not considering the role of the nurse in the handover process.
Qualitative, quantitative, and/or mixed-methods research.	
Systematic reviews or meta-analyses on the study subject.	

**Table 3:** Publications included in the review and their characteristics.

Source	Type of Study	Population	GR	LE	RB
Directly Comparing Handoff Protocols for Pediatric Hospitalists (Lazzara et al., 2016 [30])	RCT	Paediatric medical	1B	A	7.27
Changes in medical errors after implementation of a handoff program (Starmer et al., 2014 [31])	Quasi-experimental NRCT	Paediatric medical	2B	B	10
Handing Off Safety […] (Groves et al., 2016 [21])	Qualitative	Paediatric nursing	3A	1C	10
I-PASS Mentored Implementation Handoff Curriculum: […] (O’toole et al., 2019 [2])	Quasi-experimental NRCT	Paediatric medical	2B	B	8.57
I-PASS Adherence and Implications for Future Handoff Training (Hughes et al., 2019 [18])	Quasi-experimental NRCT	Paediatric medical	2B	B	10
Improving clinical handover in a paediatric ward: implications for nursing […] (Mannix et al., 2017 [32])	Quasi-experimental NRCT	Paediatric nursing	2B	B	8.57
Nursing clinical handover […] (Brown et al., 2014 [12])	Mixed	Paediatric nursing	D5	1C	9.41
Multidisciplinary handoffs improve perceptions of communication (Solan et al., 2014 [33])	Quasi-experimental NRCT	Paediatric multidisciplinary	2B	B	10
Creating a safe, reliable hospital at night handover: a case study […] (McQuillan et al., 2014 [34])	Observational	Adult/mixed	3A	1C	9.09
A tool for assessing the quality of nursing handovers: a validation study (Ferrara et al., 2017 [7])	Cross-sectional observational	Adult/mixed nursing	C4	1C	8.63
Challenges of Nursing […] (Sabet et al., 2014 [15])	Qualitative	Adult nursing	D5	1C	8
Standardized Bedside Handoff: […] (Fucik, 2019 [9])	Mixed	Paediatric nursing	3A	1C	7.64
Implementation […] of an Institution-Wide EHR-Integrated Handoff Note (Arsoniadis et al., 2022 [8])	Mixed	Adult/mixed	3A	1C	9.41
Nursing handovers and patient safety: Findings from an umbrella review (Bressan et al., 2020 [11])	Umbrella review	Adult/mixed	D5	1C	8
Impact of the communication and patient hand-off tool SBAR on patient safety: […] (Müller et al., 2018 [20])	Systematic review	Adult/mixed	B3A	1C	9
Challenges of patient handover process in healthcare services: A systematic review. (Raeisi et al., 2019 [17])	Systematic review	Adult/mixed	D5	1C	8
Diseño de un instrumento para evaluar el proceso de enlace […] (Yáñez-Corral & Zárate-Grajales, 2016 [13])	Cross-sectional observational	Adult nursing	C4	1C	9.09
Nurses’ shift reports: a systematic literature search and critical review […] (Buus et al., 2017 [14])	Systematic review	Adult nursing	D5	1C	8
Critical care nurses’ communication challenges during handovers: A systematic […] (Ahn et al., 2021 [16])	Systematic review	Adult critical care nursing	D5	1C	9

Key: GR (grade of recommendation), LE (level of evidence), RB (risk of bias in base 10), RCT (randomised clinical trial), and NRCT (non-randomised clinical trial).

**Table 4:** Standardised structured communication tools for patient handover.

Tools	Description	Observations
Flex 11 [30]	A structured tool, specific to paediatrics, with 11 categories (Demographics, Patient Summary, Current Issues, Laboratory and Other Tests, Medications, Pulm/CV/Neuro, Access, Social, As Needed, etc.).	Designed by paediatricians and intended for medical handovers. Its effectiveness was compared against SBAR.
SBAR [20]	The main method, popularised and supported by Kaiser Permanente and the Joint Commission. It is an acronym for Situation, Background, Assessment, and Recommendation.	It has been recommended and endorsed by the WHO and multiple scientific societies.
ISBAR [20,35]	Variant of SBAR that adds ‘Identify’ at the beginning, identifying the speaker and patient.	This step ensures correct identification of the patient.
ISBAR [20,35]	It is a variant of SBAR created by the Hunter-New England Area Health Service that adds ‘Introduction’ to introduce the speaker.	It arises from the initiative ‘ISBAR revisited’.
SBAR-R [20,35]	It is a variant of SBAR that adds ‘Readback’ at the end. Readback involves the recipient repeating or summarising information to confirm understanding and accuracy.	This technique helps reduce communication errors by repeating the message to the receiver.
ISBARR [20,35]	It is a variant of SBAR that adds both ‘Identify’ and ‘Readback’ at the end, thus combining the importance of correct identification with final verification.	It helps reduce communication errors by repeating the message to the recipient, in addition to highlighting identification.
iSoBAR [20,35]	The acronym stands for Identify, Situation, Observation, Background, Agreed plan, and Readback.	Initially designed for interhospital telephone handovers (X).
ISOBAR [20,35]	The acronym stands for Identify, Situation and Status, Observations, Background and History, Assessment and Actions, and Responsibility and Risk Management.	It has its own section in which the continuous assessment, severity and alert criteria are highlighted.
I-PASS [2,18]	It uses a mnemonic consisting of severity of Illness severity, Patient summary, Action list, Situation awareness and contingency planning, and Synthesis by receiver.	It is an evidence-based information transfer program. Designed by and for medical residents.
NBSR [21]	The Nursing Bedside Shift Report is a safe method endorsed by the Agency for Healthcare Research and Quality (AHRQ).	There is no paediatric adaptation or validation. It is a safer method, as it involves families.
m-ISHAPED [36]	Designed for bedside shift transfer by nurses. The acronym stands for modified ISHAPED: Introduction, Story, History, Assessment, Plan, Error Prevention, and Dialogue.	It has been tested in paediatrics in the transfer of patients between units.
SAFETIPS [33]	The standard SAFETIPS format stands for Statistics, Assessment, Focused plan, pertinent Exam findings, To dos, If/thens, Pointers/Pitfalls, and Severity of illness. It was designed for recording the on-call handover of paediatric residents in hospitalisation.	The format and its training package were designed by the Medical College of Wisconsin.

## Data Availability

No new data were created or analyzed in this study.

## Public Involvement Statement

No public involvement in any aspect of this research.

## Guidelines and Standards Statement

This manuscript was drafted against the Preferred Reporting Items for Systematic Reviews and Meta-Analyses extension for Scoping Reviews (PRISMA-ScR) guideline [23] for scoping review research.
